# Investigation of Acoustic Injection on the MPU6050 Accelerometer

**DOI:** 10.3390/s19143083

**Published:** 2019-07-12

**Authors:** Yunfan Zhang, Hui Li, Shengnan Shen, Guohao Zhang, Yun Yang, Zefeng Liu, Qisen Xie, Chaofu Gao, Pengfei Zhang, Wu Zhao

**Affiliations:** 1 School of Power and Mechanical Engineering, Wuhan University, Wuhan 430072, China; 2 Key Laboratory of Hydraulic Machinery Transients, Ministry of Education, Wuhan University, Wuhan 430072, China

**Keywords:** MEMS accelerometer, capacitor, acoustic injection, resonant frequency, deformation

## Abstract

Acoustic injection is one of the most dangerous ways of causing micro-electro–mechanical systems (MEMS) failures. In this paper, the failure mechanism of acoustic injection on the microprocessor unit 6050 (MPU6050) accelerometer is investigated by both experiment and simulation. A testing system was built to analyze the performance of the MPU6050 accelerometer under acoustic injection. A MEMS disassembly method was adopted and a MATLAB program was developed to establish the geometric model of MPU6050. Subsequently, a finite element model of MPU6050 was established. Then, the acoustic impacts on the sensor layer of MPU6050 were studied by acoustic–solid coupling simulations. The effects of sound frequencies, pressures and directions were analyzed. Simulation results are well agreed with the experiments which indicate that MPU6050 is most likely to fail under the sounds of 11,566 Hz. The failure mechanism of MPU6050 under acoustic injection is the relative shift of the capacitor flats caused by acoustic–solid resonance that make the sensor detect false signal and output error data. The stress is focused on the center linkage. MPU6050 can be reliable when the sound pressure is lower than 100 dB.

## 1. Introduction

During the past thirty years, there have been rapid developments in micro-electro–mechanical systems (MEMS) technology. As a result, the accelerometer has been miniaturized into a millimeter or even micrometer scale [1]. As a device used for measuring acceleration, the MEMS accelerometer has been widely applied in various industries, especially in the civil fields, such as mobile phones [2], civilian drones [3] and intelligent hand rings [4]. MEMS accelerometer becomes indispensable because it has the advantages of small size, extreme ruggedness, low power consumption and low price [5]. MEMS accelerometers are generally divided into two kinds: piezoresistive and capacitive-based accelerometers [6]. The primary measuring principle of capacitive-based MEMS accelerometer depends upon the changes in the capacitance via the moving plates or the sensing elements [7]. However, the MEMS accelerometer is susceptible to interference on account of its precise mechanical structures. Although the manufacturers have improved their productions, kinds of tracks can still lead to mechanical failures, such as heat, shock and sounds.

In previous studies, a series of methods have been adopted to analyze the reliability of MEMS. Khaled et al. reported an approach to analyze RF-MEMS switch failure by multi-physics simulation [8]. Wei et al. built a correlation model for monitoring data to reconstruct the sensor that failed in measurement [9]. Georg et al. proposed a kind of integration program to evaluate sensor failure [10]. Tao found that it was the fractures of the sensor structures that resulted in mechanical failures when the MEMS accelerometer was under high-frequency shocks [11]. Pradeep et al. studied the reliability of MEMS accelerometer in mechanical shocks by modeling simulations [12]. Tu et al. adopted Kriging approximation and probabilistic algorithms method with finite element simulations to study the effect of input uncertainties on the response of the MEMS structure [13]. Khanna et al. found that MEMS failure takes place between the deposited layers and this failure may occur both because of processing defects or high interfacial stress levels [14], such as a large stress gradient and mechanical shocks. Lucibello measured RF-MEMS switches with thermal and mechanical cycles, which indicated that the deformations, softening and cracks would occur on the MEMS membrane [15].

Acoustic injection is one of the most dangerous methods for disabling MEMS. Yan et al. found that the gyroscopes of phones were sufficiently sensitive to sound, which could be exploited to measure acoustic signals [16]. Son et al. found that acoustic interference could attack MEMS gyroscopes by causing denials of output [17]. Soobramaney et al. applied acoustic dampening materials on the MEMS surface to defend against acoustic interference [18]. The microprocessor unit 6050 (MPU6050) is the research object in this paper. It is the first si*x*-axis motion tracking device in the world which is composed of a three-axis accelerometer and a three-axis gyroscope [19]. The structure of MPU6050 can be simply divided into five layers: package (Quad Flat No-lead, QFN-24), cap layer, sensor layer, application-specific integrated circuit (ASIC) and lead frame.

In this paper, acoustic injection experiments were conducted, and acoustic-solid coupling simulations were performed to study the invalidation mechanism of the MPU6050 accelerometer. A testing system was built to study the acoustic influence on the performance of the accelerometer. A MEMS disassemble method was adopted to obtain the internal structures of MPU6050. Furthermore, an optical microscope was used to take micrographs of the accelerometer Y-component of MPU6050 (MPU6050-Y) from which a MATLAB program was adopted to measure the dimensions of the structures. After the full-size geometric model was established, the finite element model was established by considering the boundary conditions of fixed support, acoustic absorption and impedance. Then, a multi-physics coupling finite element simulation was performed to analyze the stress and the micro deformation of MPU6050 under different sound pressures, frequencies and directions.

## 2. Experiment Investigation

**Figure 1:**
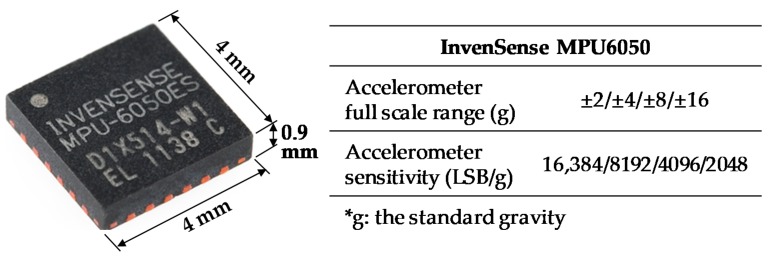
InvenSense microprocessor unit 6050 (MPU6050) and its main parameters [19].

**Figure 2:**
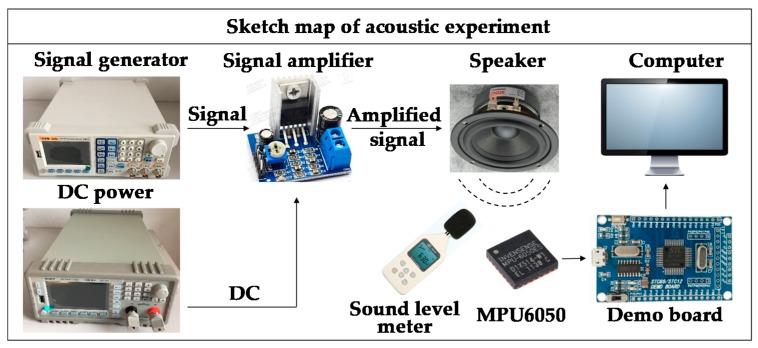
Overview of the sound injection experiment.

An experiment of sound injection on MPU6050 was carried out to study the acoustic influence on the output of the accelerometer. Figure 1 shows the parameters of InvenSense MPU6050. Its dimension is 4.0 × 4.0 × 0.9 mm. The accelerometer part is programmable to full-scale range of ±2 g, ±4 g, ±8 g, or ±16 g. Correspondingly, the sensitivity is 16,384 LSB/g, 8192 LSB/g, 4094 LSB/g or 2048 LSB/g. Figure 2 is an overview of the sound injection experiment. A signal generator was used to generate electrical signals with variable frequencies from 0 Hz to 12,000 Hz. A DC (direct current) power was used to supply the speaker with voltage from 6 V to 12 V. A signal amplifier was used to amplify the electrical signals and transfer amplified signals into the speaker. A demo board was used to obtain acceleration data from MPU6050. A sound level meter was used for real-time monitoring of the sound level at the position of MPU6050 which was put 5 cm under the speaker.

**Figure 3:**
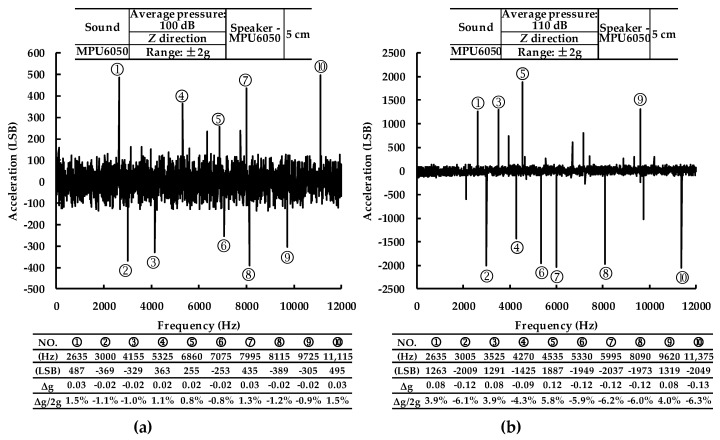
The outputs of *Y*-axis accelerometer by (**a**) 100 dB acoustic wave; (**b**) 110 dB acoustic wave.

After being set to the full-scale range of ±2 g, MPU6050 was subjected to acoustic waves whose frequency varied from 0 Hz to 12,000 Hz and the sound pressure was in an average of 100 dB or 110 dB. At the meantime, the single chip computer sampled the outputs of MPU6050-Y every 5 Hz which was shown in Figure 3. MPU6050 should output zero when there is no velocity change. It could be found that most of the outputs were under ±200 LSB but there were significant increases in several specific frequencies. When the average sound pressure was 100 dB, the frequencies at which the significant increases happened were 2635 Hz, 3000 Hz, 4155 Hz, 5325 Hz, 6860 Hz, 7075 Hz, 7995 Hz, 8115 Hz, 9725 Hz and 11,115 Hz. When the average sound pressure was 110 dB, the frequencies were 2635 Hz, 3005 Hz, 3525 Hz, 4270 Hz, 4535 Hz, 5330 Hz, 5995 Hz, 8090 Hz, 9620 Hz and 11,375 Hz. Moreover, the accelerometer output of MPU6050-Y reached a maximum of 0.03 g and −0.13 g when the average sound pressure was 100 dB and 110 dB respectively.

## 3. Modeling and Simulation

The interference of the sound wave on MPU6050 performance was a typical acoustic–solid coupling issue. ANSYS (Canonsburg, PA, USA) has an advantage in multi-physics field simulation for which it has been used in this research. The simulation can be divided into four steps: disassembling MPU6050, establishing the geometric model, establishing the finite element model and performing coupling simulation.

### 3.1. Disassembly of MPU6050

MPU6050, especially its sensor layer, has a compact structure. Therefore, MPU6050 was disassembled to establish a precise geometrical model. MPU6050 was covered with epoxy resin which is a kind of thermolabile material. By using a hot wind gun (Lo’ Master 6512) to heat MPU6050, the epoxy resin shell was oxidized and the package became exfoliating. Following the clean-up, a process of corroding the adhesives between cap wafer and ASIC by using hydrochloric acid (HCl) was taken to separate the cap wafer and ASIC. Then, the sensor layer was visible.

### 3.2. Geometric Model

**Figure 4:**
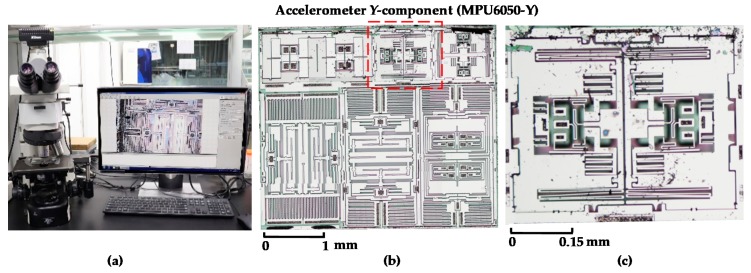
(**a**) Nikon microscope for taking the micrograph of sensor layer; (**b**) micrograph of sensor layer; (**c**) partially enlarged micrograph of MPU6050-Y.

After the disassembly process, an optical microscope (Nikon Eclipse Ci-L, Figure 4a) was used to take the micrograph of the sensor layer. Figure 4b shows the micro structures of sensor layer which consisted of six independent areas. The upper three parts were the three-axis accelerometer and the lower three parts were the three-axis gyroscope. Figure 4c is the enlarged picture of MPU6050-Y which was the key research object in this paper.

A MATLAB (Natick, MA, USA) program was developed to extract the dimensions of MPU6050-Y from the above micrograph. To decrease the image noise, Gauss filter was used for micrograph pretreatment after grey processing. The gradient vectors of the grey level of images change substantially on the edges but little away from the edges. Therefore, the gradient vectors were detected to determine whether there was a structure edge and located its position. Then, double-threshold detection method was adopted to determine the true outline. Finally, by comparing with the reference dimension, the actual dimensions of MPU6050 were obtained. This method had been calibrated by measuring the standard length and results showed that the accuracy was beyond 99%.

**Figure 5:**
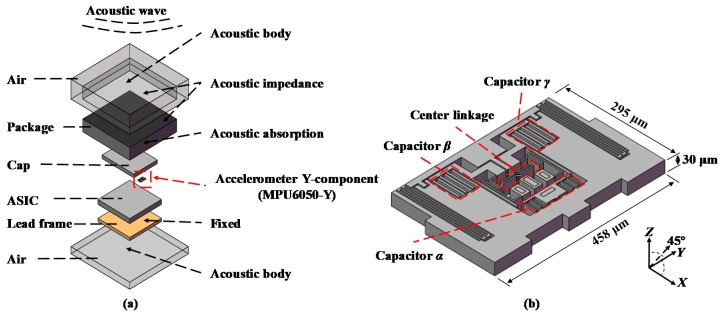
(**a**) Schematic of the MPU6050 model; (**b**) geometric model of MPU6050-Y.

Solidworks 2016 (Vélizy-Villacoublay, France) was used to establish the full-scale geometric model. To reduce the computation cost, a few steps were made to simplify the model: (1) Removing the pins on the bottom, the ASIC circuit and the sensor layer structures except for MPU6050-Y. (2) As MPU6050-Y is a symmetrical structure (Figure 4c), only the right part was established in the geometric model. Figure 5a shows the main structures of MPU6050, which includes package, cap layer, sensor layer, ASIC and lead frame. Figure 5b shows the details of MPU6050-Y whose dimension was 458 μm × 295 μm × 30 μm. As marked out in Figure 5b, its functional units were three independent flat capacitor groups: capacitor α (composed of five short parallel flat capacitors), capacitor β and capacitor γ (each composed of four long parallel flat capacitors).

Propagation of sound wave in different media is calculated as

(1) $$P_{L}=20\mathrm{lg}\frac{\alpha \sqrt{I\rho S}}{P_{0}}$$

where *P*_L_ is the sound pressure level, *α* is the absorption coefficient of the medium, *I* is the acoustic intensity, *ρ* is the density of the medium, *S* is the acoustic speed in the medium and *P*_0_ is the reference sound pressure. The capacitance of the parallel flat capacitor is calculated as

(2) $$C=\frac{\varepsilon A}{d}$$

where ε is the permittivity, *A* is the positive area of the capacitor flats, *d* is the thickness of the gap between the flats. The change of capacitance is calculated as

(3) $$\Delta C=\varepsilon_{0}\left(\frac{A_{0}}{d_{0}}-\frac{A_{1}}{d_{1}}\right)$$

where $\varepsilon_{0}$ is the permittivity of vacuum which is equal to 8.85 × 10^−12^ F/m, *A*_0_ is the initial positive area of the capacitor flats, *d*_0_ is the initial thickness of the gap between the flats, *A*_1_ is the positive area of the capacitor flats and *d*_1_ is the thickness of the gap between the flats when the capacitor has maximum deformation.

### 3.3. Finite Element Model

**Figure 6:**
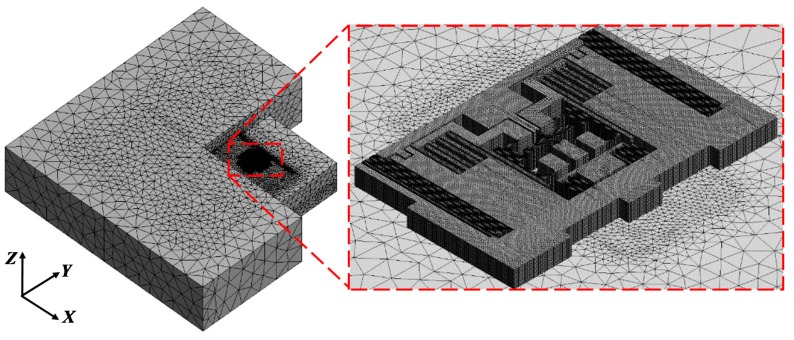
Finite element model of MPU6050.

Figure 6 shows the finite element model of MPU6050. The left part is the general view of the model and the right part is a magnification of the refined mesh area. A multi-scale mesh was used to reduce the computation cost because of the structural complexity with both micron scale and millimeter scale. In particular, the mesh of MPU6050-Y was locally refined to the size of 1 μm. A total of 2,368,244 mesh elements and 5,768,061 nodes were generated in the entire simulation domain.

The physical properties of each component of MPU6050 are listed in Table 1. The simulation boundary conditions are shown in Figure 5a and Table 2. Two air layers were set to acoustic bodies as the medium for acoustic transmission between the acoustic source and MPU6050. There was energy absorption and loss when sound passed through the media, especially in the interface between two layers. Therefore, the acoustic absorption and the impedance coefficient of air and package were considered in the finite element analysis. The acoustic impedance of air is 409.4 Pa·s/m. The acoustic absorption coefficient and the acoustic impedance of package material were set as 0.15 and 931,000 Pa·s/m. The lead frame was fixed similar to that on a circuit board in real situations. The acceleration due to gravity was set to the standard value of 9.8 m/s^2^. Sound waves with different pressures, frequencies and directions were applied on MPU6050 as the input in the simulation. The external loads were sounds with different parameters. In detail, sound frequency was set to the four mode frequencies of MPU6050. Sound pressure was set to 94 dB, 100 dB, 108 dB and 114 dB which are typical environmental sound pressures. Sound direction was set to four angles: *X*, *Y*, *Z* and 45° which are very representative.

The acoustic–solid coupling simulation of MPU6050 was performed by ANSYS 16.0. In detail, the modal module was used for modal analysis and the harmonic module was used to study the effect of various sounds. A workstation (DELL Precision T7910) was used for the simulation and each case took approximately 14 h.

### 3.4. Simulation Results and Discussion

(A) Modal Simulation of MPU6050

**Figure 7:**
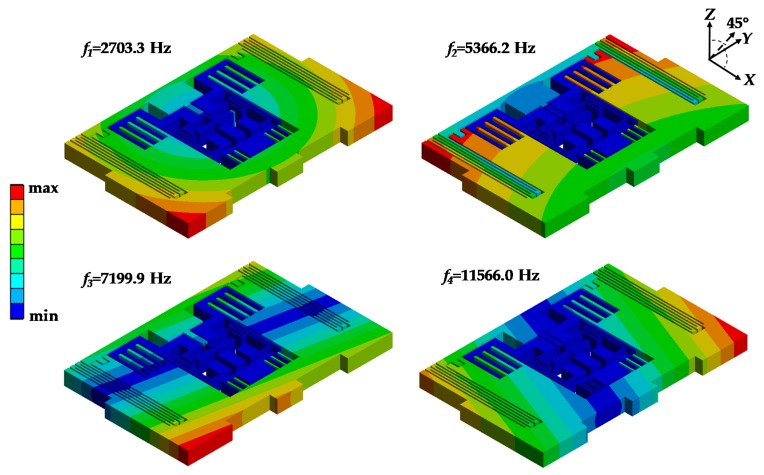
Deformation contours of MPU6050-Y in the first four modes.

A modal simulation was performed to obtain the natural frequencies of MPU6050-Y. The first four modes of MPU6050-Y were 2703.3 Hz, 5366.2 Hz, 7199.9 Hz and 11,566.0 Hz which are well agreed with the resonant frequencies measured by experiment (Table 3). The deviations between simulation and experiment results were less than 4% in most cases. The deformations of MPU6050-Y in the different modes are shown in Figure 7. It could be found that the deformations of mode 1 and mode 2 were distributed in a ring form with the maximum located in the *X*-axis corners. The deformations of mode 3 and mode 4 were distributed in a strip form with the maximum located on the *Y*-axis corners. The minimum deformations of all the four modes were located in the central part.

(B) Effects of Sound Frequencies and Pressures

**Figure 8:**
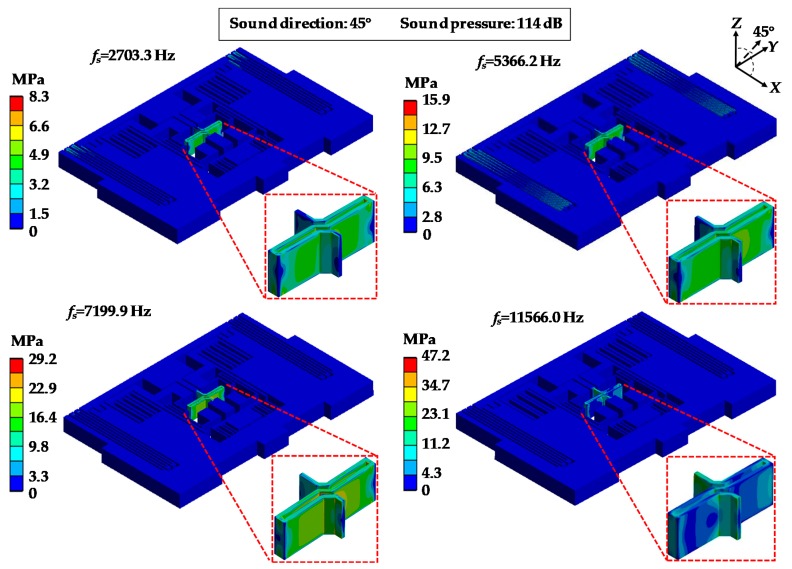
Equivalent stress distributions of MPU6050-Y under acoustic injection of different sound frequencies.

**Figure 9:**
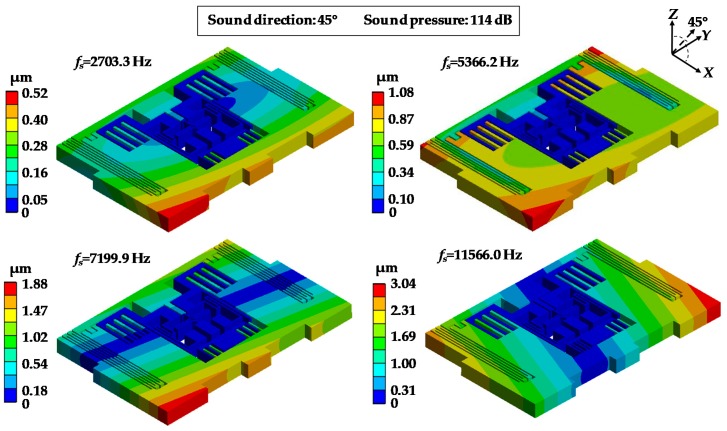
Deformation distributions of MPU6050-Y under acoustic injection of different sound frequencies.

Figure 8 and Figure 9 show the distribution of the equivalent stress and deformations of MPU6050-Y under an acoustic injection of resonant frequencies while the acoustic source was in the direction of 45° and the sound pressure was 114 dB. From Figure 8, it can be observed that the stresses were concentrated in the central linkage and the maximum stresses were 8.3 MPa, 15.9 MPa, 29.2 MPa and 47.2 MPa. Figure 9 shows that the deformations were distributed in different forms, which were similar to the modal and the maximum values were 0.30 μm, 0.62 μm, 1.88 μm and 3.04 μm.

**Figure 10:**
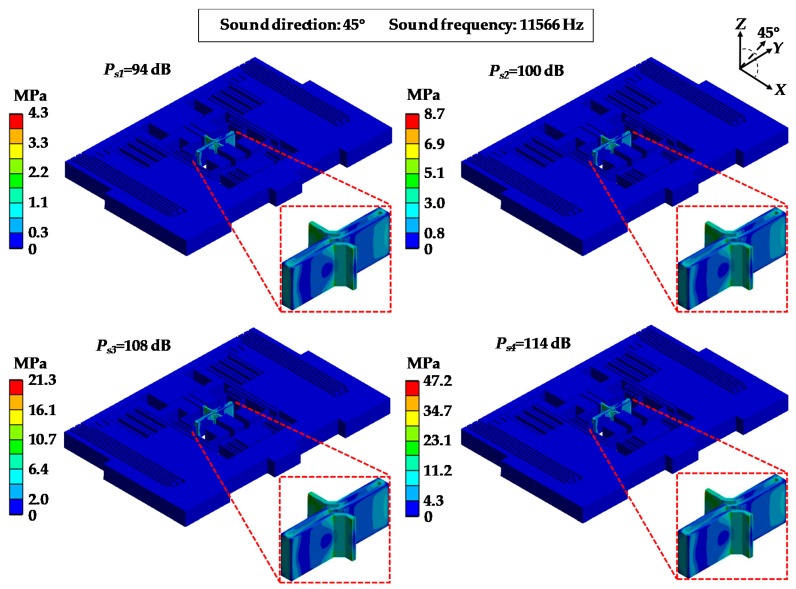
Equivalent stress distributions of MPU6050-Y under acoustic injection of different sound pressures.

**Figure 11:**
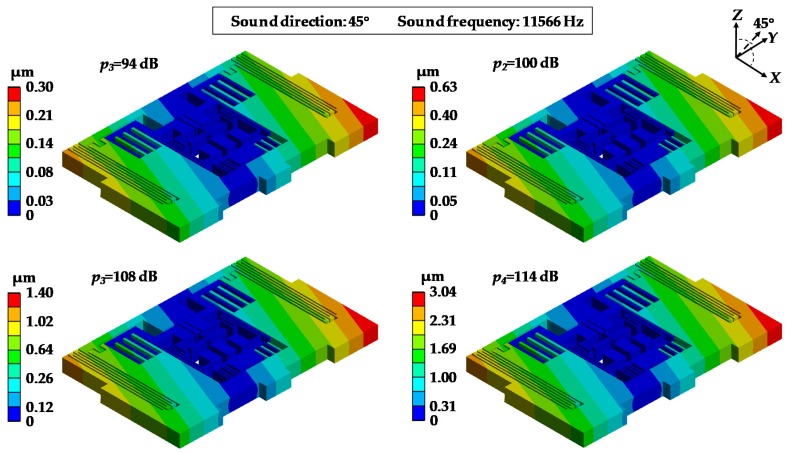
Deformation distributions of MPU6050-Y under acoustic injection of different sound pressures.

Figure 10 and Figure 11 show the distribution of the equivalent stress and deformations of MPU6050-Y under acoustic injections of different sound pressures as 94 dB, 100 dB, 108 dB and 114 dB when the sound frequency was 11,566 Hz which equals mode 4 of MPU6050 (Table 3). In Figure 10, it can be observed that the stresses were distinctly concentrated in central bar linkage and the maximum stresses were 4.3 MPa, 8.7 MPa, 21.3 MPa and 47.2 MPa. Figure 11 shows the deformations of MPU6050-Y and the maximum deformations were 0.52 μm, 1.08 μm, 1.40 μm and 3.04 μm.

**Figure 12:**
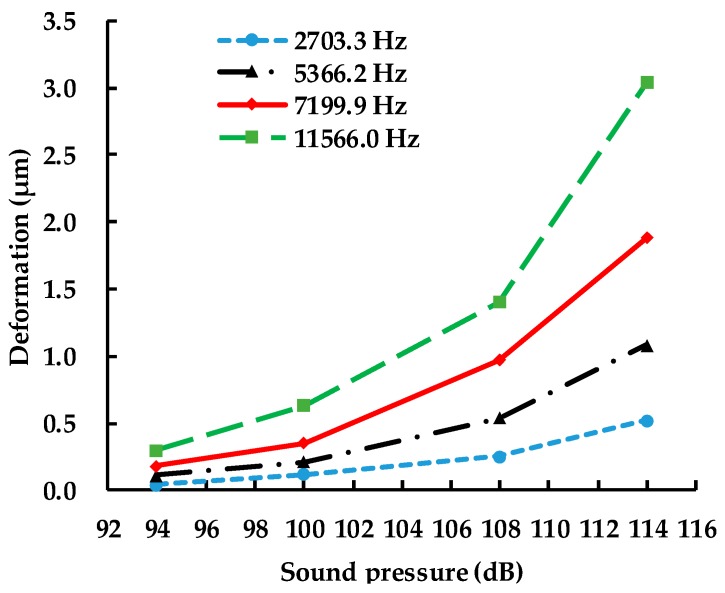
Maximum deformations of MPU6050-Y under acoustic injection of different sound frequencies and pressures.

The features of the deformations of MPU6050-Y under different sound pressures and frequencies are shown in Figure 12. It can be found that the sound pressure has a great effect on MPU6050-Y because the deformation values enlarge with increasing amplitude of sound pressure. When the acoustic wave was set to 114 dB and 11,566 Hz, the deformation of MPU6050-Y reached a maximum of 3.04 μm. According to the deformations of capacitors, the maximum change of MPU6050-Y capacitance was 11.16% (Equation (3)).

The above results indicate two invalidation mechanisms of MPU6050 under acoustic injection. One is the relative shift of the capacitor flats that cause capacitance changes and the other is the fatigue crack on the central linkage. The sensor structures are hung above ASIC layer with several micro pillars fixed to the bottom in a vacuum cell, which make the pillars the only way for sound energy transmission. The sensor frame is linked with central pillars by elastic beams that make the frame sensitive to vibration. When the resonant-frequency sound was applied on MPU6050, the sound energy transmitted to sensor layer through the pillars. This transmission caused a relative shift in the capacitor flats, leading to changes in the positive area and the capacitor gaps. Then, the sensor outputted error data. While the vibration occurred, the main stress was concentrated on the central linkage where the fatigue crack would most probably emerge.

(C) Effects of Sound Directions

**Figure 13:**
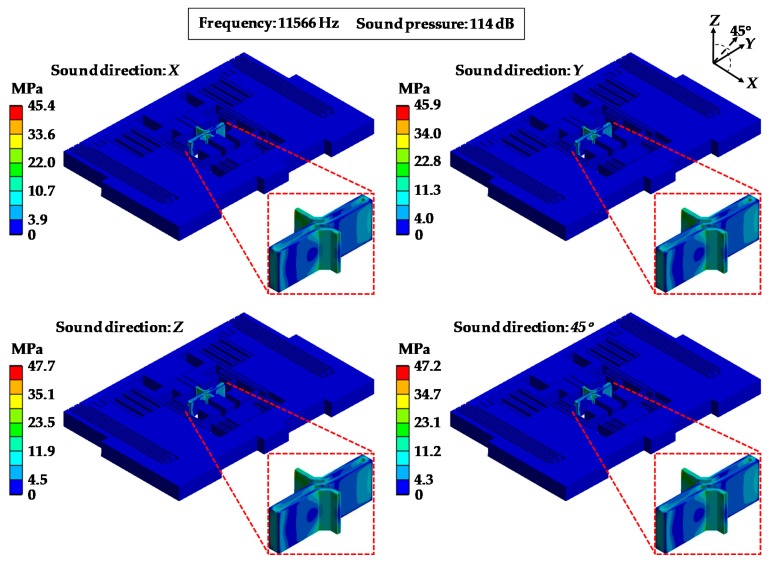
Equivalent stress of MPU6050-Y under acoustic injection in different sound directions.

**Figure 14:**
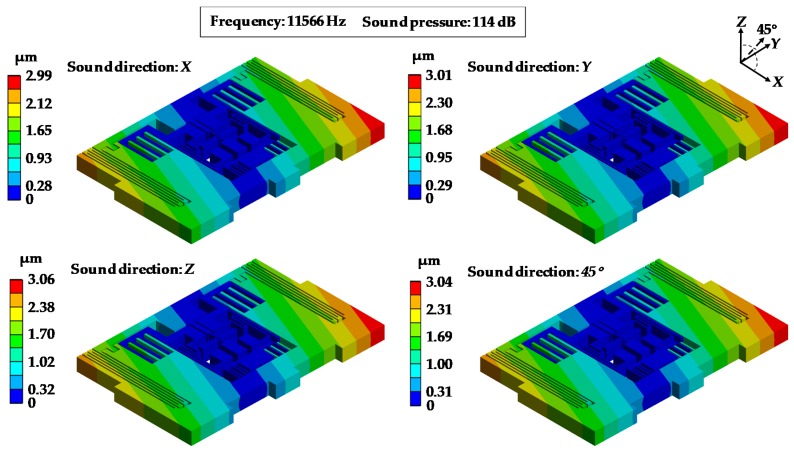
Deformations of MPU6050-Y under acoustic injection in different sound directions.

Figure 13 and Figure 14 show the distributions of the equivalent stress and the deformations of MPU6050-Y under acoustic injection from four directions (*X* axis, *Y* axis, *Z* axis and 45°) when the sound frequency was 11,566 Hz and the sound pressure was 114 dB. It can be seen that the stresses were concentrated on the central part (Figure 13) and the maximum deformation was located in the corner (Figure 14). MPU6050-Y reaches the maximum stress of 47.7 MPa and maximum deformation of 3.06 μm under the *Z* axis direction sound.

Table 4 shows the maximum deformations of MPU6050-Y under different sound directions when the sound pressure was 114 dB. It shows that the maximum deformations varied little with changes of the sound direction. When the acoustic source was applied in the *Z*-axis direction, the deformations reached a maximum in each direction.

## 4. Conclusions

In this paper, a testing system was built to study the acoustic influence on the performance of the MPU6050 accelerometer. A MEMS disassemble method was adopted to obtain the structures of MPU6050 and a MATLAB program was developed to measure the structural dimensions from the micrograph taken by an optical microscope. A finite element model was established considering the exact geometric dimensions and boundary conditions. Then, an acoustic–solid coupling simulation was performed to study the failure mechanism of the MPU6050 accelerometer. The simulation results agreed well with the experiment results. It has low deviations between the resonant frequencies of experiments and simulation modes which are less that 4% in most cases.

The conclusion can be given as follows: (1) It indicated the failure mechanism of MPU6050 under acoustic injection that the relative shift of the capacitor flats caused by acoustic–solid resonance makes the sensor detect false signal and output error data. (2) When the sound is in the fourth resonant frequency of MPU6050 (i.e., 11,566 Hz), the interference on sensor was most serious. (3) It iss suggested for hardware design that the intensity of linkage should be improved to reduce the influence of stress concentration. (4) Results indicate that the deformations of MPU6050-Y are in low values when the sound pressure is less than 100 dB which means MPU6050 can remain reliable within the interval of 0 to 100 dB.

## Figures and Tables

**Table 1:** Physical properties of MPU6050.

Structure	Material	Property (Unit)	Value	Reference
Package	Epoxy resin	Density: *ρ*_1_ (kg/m^3^)	980	[20]
		Young’s modulus: E1 (GPa)	2.89	[20]
		Poisson ratio: *v*_1_	0.40	[20]
MEMS cap ASIC Accelerometer	Silicon	Density: *ρ*_2_ (kg/m^3^)	2330	[21]
		Young’s modulus: E2 (GPa)	150	[21]
		Poisson ratio: *v*_2_	0.22	[21]
Lead frame	Alloy 42	Density: *ρ*_3_ (kg/m^3^)	8150	[22]
		Young’s modulus: E3 (GPa)	145	[23]
		Poisson ratio: *v*_3_	0.30	[23]

**Table 2:** Parametric values of variables used in the simulation.

Property (Unit)	Value	Reference
Air density: *ρ_a_* (kg/m^3^)	1.225	[24]
Pressure of acoustic wave: *p_s_* (Pa)	1/2/5/10	
Frequency of acoustic wave: *f_s_* (Hz)	2703.3/5366.2/ 7199.9/11,566	
Distance between acoustic source and MPU6050: L (cm)	5	
Direction of acoustic wave	x/y/z/45°	
Gravity: *G* (m/s^2^)	9.8	
Acoustic absorption coefficient of epoxy resin: *α_er_*	0.15	[25]
Acoustic impedance of air: *Z_a_* (Pa·s/m)	409.4	[26]
Acoustic impedance of epoxy resin: *Z_er_* (Pa·s/m)	931,000	[26]

**Table 3:** Comparison of simulation and experiment results.

**Simulation**	**Mode 1**		**Mode 2**		**Mode 3**		**Mode 4**	
	2703.3 Hz		5366.2 Hz		7199.9 Hz		11,566.0 Hz	
**100 dB experiment**	**NO. 1**	**Δ**	**NO. 4**	**Δ**	**NO. 6**	**Δ**	**NO. 10**	**Δ**
	2635 Hz	−2.5%	5325 Hz	−0.8%	7075 Hz	−1.7%	11,115 Hz	−3.9%
**110 dB experiment**	**NO. 1**	**Δ**	**NO. 6**	**Δ**	**NO. 8**	**Δ**	**NO. 10**	**Δ**
	2635 Hz	−2.5%	5330 Hz	−0.7%	8090 Hz	12.4%	11,375 Hz	1.7%

**Table 4:** Maximum deformations of MPU6050-Y under acoustic injection in different sound directions.

Amplitude (dB)	Frequency (Hz)	Maximum Deformation (μm)			
		*X*	*Y*	*Z*	45°
114	2703.3	0.51	0.52	0.54	0.52
	5366.2	1.06	1.09	1.10	1.08
	7199.9	1.85	1.87	1.90	1.88
	11,566.0	2.99	3.01	3.06	3.04
